# Divergent long-term trends in semen quality and reproductive hormones across the COVID-19 pandemic era in infertile men: an age-stratified retrospective study

**DOI:** 10.1186/s12610-026-00309-1

**Published:** 2026-03-26

**Authors:** Peilian Li, Shuang Liu, Ke Chen, Yang Gao

**Affiliations:** 1 Reproductive Medicine Center, Chongqing Health Center for Women and Children (Women and Children’s Hospital of Chongqing Medical University), Chongqing, China; 2 Department of the Assisted Reproduction, The Affiliated Hospital, Southwest Medical University, Luzhou, Sichuan China

**Keywords:** COVID-19 pandemic, Male infertility, Semen analysis, Reproductive hormones, Longitudinal study, Age effects, Recovery trajectories, Generalized estimating equations, Pandémie de COVID-19, Infertilité masculine, Analyse du sperme, Hormones reproductives, Étude longitudinale, Effets de l’âge, Trajectoires de Récupération, Équations d’Estimation généralisées

## Abstract

**Background:**

Male reproductive dysfunction has been reported during the acute phase of COVID-19. However, the long-term patterns in semen quality and hormonal parameters across the extended pandemic and post-pandemic periods, and whether these patterns differ by age, remain poorly characterized. This large-scale, retrospective study was conducted to delineate these trajectories in infertile men.

**Results:**

Among 90,125 records, normal sperm morphology showed a sustained deficit: the proportion of men with morphology ≥ 4% declined from 88.9% pre-pandemic to 55.1% in Post-Phase 2 (*p* < 0.001), with nearly half falling below the WHO clinical threshold (4%). Serum testosterone decreased modestly from 4.46 to 4.19 ng/mL (6.1% reduction; (*p* = 0.006) but remained above the hypogonadism threshold for most men. These alterations persisted two years into the post-pandemic period without returning to pre-pandemic baselines.

**Conclusions:**

The COVID-19 pandemic was associated with persistent, clinically meaningful deficits in normal sperm morphology and a moderate, non-hypogonadal testosterone suppression, with distinct age-dependent patterns. These findings underscore the need for age-stratified reproductive health surveillance following major population-level stressors.

**Supplementary Information:**

The online version contains supplementary material available at 10.1186/s12610-026-00309-1.

## Résumé

**Contexte:**

Un dysfonctionnement reproductif masculin a été signalé durant la phase aiguë du COVID-19. Cependant, les tendances à long terme de la qualité du sperme et des paramètres hormonaux à travers les périodes prolongées de pandémie et de postpandémie, ainsi que la différence de ces tendances selon l’âge, restent peu caractérisées. Cette étude rétrospective à grande échelle a été menée pour délimiter ces trajectoires chez les hommes infertiles.

**Résultats:**

Parmi 90 125 dossiers, la morphologie normale des spermatozoïdes a montré un déficit soutenu : la proportion d’hommes avec une morphologie ≥4 % est passée de 88,9 % avant la pandémie à 55,1 % en post-phase 2 (p < 0,001), près de la moitié se situant en dessous du seuil clinique de l’OMS (4 %). La testostérone sérique a légèrement diminué de 4,46 à 4,19 ng/mL (réduction de 6,1 % ; p = 0,006), mais elle restait au-dessus du seuil d’hypogonadisme chez la plupart des hommes. Ces modifications ont persisté deux ans après le début de la période postpandémique sans revenir aux niveaux de base d’avant la pandémie.

**Conclusions:**

La pandémie de COVID-19 a été associée à des déficits persistants et cliniquement significatifs de la morphologie normale des spermatozoïdes ainsi qu’à une suppression modérée de la testostérone, mais non hypogonadique, avec des schémas distincts en fonction de l’âge. Ces résultats soulignent la nécessité d’une surveillance de la santé reproductive stratifiée par âge à la suite des principaux facteurs de stress au niveau de la population.

## Background

Global health crises, such as the SARS-CoV-2 pandemic, can induce profound and persistent perturbations through a combination of direct viral effects and indirect pandemic-related stressors (e.g., psychological distress, lifestyle changes, and healthcare disruptions). The male reproductive system may be particularly susceptible, raising concerns about long-term population fertility given that male factors contribute to ~ 50% of global infertility cases. Although direct viral involvement remains plausible, supported by rare reports of SARS-CoV-2 RNA detected in semen and potential mechanisms of testicular injury via ACE2/TMPRSS2 receptors [1–5], population-level studies must also account for the broader impact of the pandemic environment. Consistent with this, accumulating clinical evidence has documented significant impairments in semen quality and reproductive hormones during the pandemic period [6–9], underscoring the need to clarify long-term trajectories and recovery patterns following such a widespread, multifaceted stressor.

However, current evidence on male reproductive function during the COVID-19 era reveals conflicting findings, ranging from significant short-term deficits in semen quality [6] to rapid recovery [7, 8] or no observable effect [9]. This inconsistency points to three critical research gaps. First, longitudinal studies with pre-pandemic baseline data are scarce, hindering robust inference about recovery relative to historical norms. Second, beyond conventional semen values indices, functional and mechanistic biomarkers, including sperm acrosomal function, seminal plasma biochemistry, and comprehensive hormonal profiling, have been insufficiently investigated in this context. Third and most importantly, the long-term recovery patterns of specific reproductive parameters across extended post-pandemic phases remain poorly characterized. Moreover, although age is known to modify vulnerability to diverse health stressors and Covid [10], its role in shaping long-term temporal trajectories of male reproductive recovery remains completely unexplored.

To address these gaps, we analyzed an eight-year longitudinal dataset from infertile men, stratifying records into a pre-pandemic period, a pandemic period, and two post-pandemic periods. This study was designed to compare temporal trends rather than individual SARS-CoV-2 infection status; thus, observed changes may reflect the combined influence of viral infection, pandemic-associated stressors, and other temporal factors. For routine semen parameters (sperm concentration, motility, and total count), longitudinal comparisons with the pre-pandemic period were precluded owing to a methodological transition from manual counting to CASA (Computer-Assisted Semen Analysis); therefore, trend analyses for these parameters were restricted to the three post-transition periods.

Specifically, this study aimed to: (1) delineate temporal trends in semen quality (with methodological caveats for routine parameters), seminal plasma biochemistry, and reproductive hormones across pandemic phases; (2) distinguish parameters that returned to baseline from those that remained below baseline; and (3) determine how age modulates these recovery dynamics. Collectively, our findings provide essential evidence for implementing age-stratified clinical monitoring and long-term reproductive health surveillance in the aftermath of global health emergencies, while also contributing to the understanding of how major population-level stressors differentially impact male reproductive resilience.

## Men and methods

### Study design and population

**Fig. 1:**
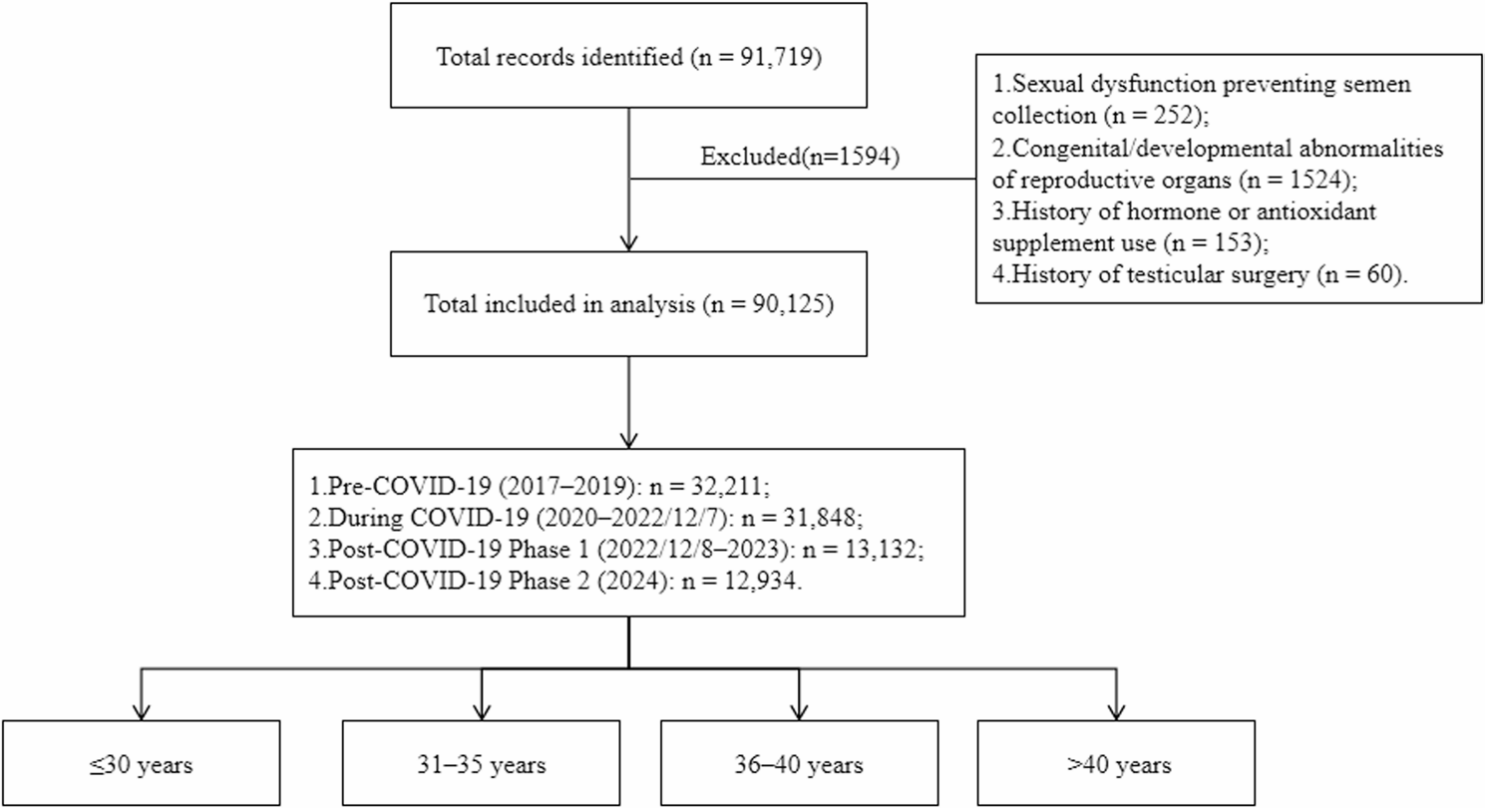
Participant flow diagram. The diagram illustrates the selection process of the study population. A total of 91,719 patient records were initially screened. After applying the exclusion criteria, 90,125 men were included in the final analysis. The numbers of participants in each pandemic period are shown. Age stratification was performed within each period. Abbreviations: COVID-19, coronavirus disease 2019

This retrospective, observational study analysed semen paramaters and serum reproductive hormone measurements from infertile patients attending the Reproductive Medicine Center, Chongqing Maternal and Child Health Hospital, between January 2017 and December 2024. The analytical framework was designed to compare trends across predefined pandemic periods rather than to evaluate outcomes by individual SARS-CoV-2 infection status. The following patients were excluded: those with sexual dysfunction preventing semen sample collection; individuals with congenital or developmental abnormalities of the reproductive organs; patients with a history of hormone or antioxidant supplement use that could affect spermatogenesis; and those with a history of testicular surgery (e.g., cryptorchidism, hydrocele, testicular torsion, or testicular tumors). A participant flow diagram is presented in Fig. 1.

### Study periods and grouping

On the basis of the chronology of the COVID-19 pandemic and major public health policy shifts in China, the study samples were categorized into four consecutive periods:

1. Pre-COVID-19: January 1, 2017 – December 31, 2019.
2. During COVID-19: January 1, 2020 – December 7, 2022 (peak pandemic phase under stringent control policies).
3. Post-COVID-19 Phase 1: December 8, 2022 – December 31, 2023 (immediate period following the major policy adjustment).
4. Post-COVID-19 Phase 2: January 1, 2024 – December 31, 2024 (extended recovery phase).

Within each period, participants were further stratified into four age groups: ≤30 years, 31–35 years, 36–40 years, and > 40 years.

### Assessed parameters

The following outcomes were evaluated:

- Routine semen parameters: Semen volume, sperm concentration, total sperm count, progressive motility (%), non-progressive motility (%), and immotile spermatozoa (%).
- Sperm morphology: Percentage of spermatozoa with normal morphology and the proportion of samples with normal sperm morphology ≥ 4%.
- Acrosomal function: Acrosome reaction percentage (AR).
- Seminal plasma biochemistry: Fructose, zinc, and neutral α-glucosidase levels.
- Reproductive hormones: Serum estradiol (E2), follicle-stimulating hormone (FSH), luteinizing hormone (LH), and testosterone (T).
- Immunological parameter: Anti-sperm antibody (ASA) positive/negative percentage.

### Laboratory procedures

All laboratory assays were performed by experienced technicians following standardized protocols, with routine internal quality control implemented throughout the study period.

Methodological Consistency and a Key Transition.

For data comparability, it is important to note that only routine semen parameters (sperm concentration, total count, and motility) underwent a methodological transition during the study period. All other assessed parameters (sperm morphology, acrosomal function, seminal plasma biochemistry, reproductive hormones, and immunological assay) were measured using the same laboratory methods and protocols throughout the entire study period (2017–2024).

### Semen collection and routine analysis

Semen samples were obtained by masturbation after 2–7 days of sexual abstinence. After liquefaction at 37 °C for 30 min, semen volume was recorded by the weighing method, in accordance with the WHO Laboratory Manual (5th edition). For routine semen parameters (sperm concentration, progressive motility, non-progressive motility, immotility), samples from the Pre-COVID-19 period were analyzed by manual counting, while samples from the During COVID-19 period onward were analyzed using a Computer-Assisted Semen Analysis (CASA) system (JingCheng, JiangSu, China). To ensure methodological consistency, statistical comparisons for sperm concentration, motility, and total sperm count (calculated as semen volume × sperm concentration) were restricted to the three periods using CASA methodology (During, Post-Phase 1, and Post-Phase 2). Semen volume was also analyzed within these same three periods to maintain uniformity in the dataset. Sperm morphology was assessed after DIFF-quick staining (Kit from Sperm Morphology Quick Stain, BASO, Zhuhai, China).

### Assessment of other parameters

Anti-sperm Antibody Testing: Performed using a direct mixed antiglobulin reaction (MAR) test (BRED Life Science Technology Inc., Shenzhen, China). A result was positive if ≥ 50% of motile spermatozoa were bound by latex particles.

Sperm Acrosomal Function: The acrosome reaction was induced by incubating spermatozoa collected by swim-up with 10 µM calcium ionophore (Cellpro Biotech, Zhejiang, China) for 15 min at 37 °C. Acrosomal status was assessed by flow cytometry after staining with fluorescein isothiocyanate-labeled Pisum sativum agglutinin (FITC-PSA). The acrosome reaction was defined as the spermatozoa exhibiting low FITC fluorescence (acrosome-reacted) and calculated as a percentage, strictly following the manufacturer’s instructions.

Seminal Plasma Biochemistry: Liquefied semen was centrifuged at 3000 g for 10 min to obtain plasma. Fructose (enzymatic method, BRED Life Science Technology Inc., Shenzhen, China), zinc (colorimetric method, Rajbio, Tianjin, China), and Neutral α-glucosidase activity was measured using the modified Cooper method (BRED Life Science Technology Inc., Shenzhen, China) under conditions that inhibit acid α-glucosidase, strictly following the manufacturer’s instructions.

Hormone Analysis: Serum levels of E2, FSH, LH, and T were quantified using commercial chemiluminescent immunoassay kits (Abbott Ireland Diagnostics, Longford Ireland).

### Statistical analysis

All statistical analyses were performed using SPSS (Version 29.0). Continuous variables are presented as median (IQR). For parameters measured consistently across all periods, comparisons were made across the four periods using the Kruskal-Wallis test with Bonferroni-corrected pairwise comparisons where significant.Categorical variables were compared using the Chi-squared test.

To account for repeated measurements from the same individuals, we employed Generalized Estimating Equations (GEE) with an exchangeable correlation structure. The primary independent variable was the categorical “pandemic period” (During, Post-Phase 1, Post-Phase 2).

The choice of distribution family and link function in the GEE was tailored to each outcome variable’s data characteristics (see table footnotes for Supplementary Table 3). Owing to a methodological transition from manual counting to CASA for routine semen parameters, analyses for sperm concentration, motility, and total count were restricted to the three CASA-consistent periods (During, Post-Phase 1, Post-Phase 2) to eliminate confounding effects of methodological change.

To evaluate the modifying effect of age, we included a time period × age group interaction term in the models. No additional covariates were included, as the primary aim was to delineate population-level temporal trends associated with the pandemic period as a composite exposure. A P-value < 0.05 was considered statistically significant.

## Results

### Study population and overall temporal trends

This retrospective analysis initially encompassed 90,125 patient records. Following the application of exclusion criteria (see Fig. 1 for flowchart), data were analyzed across four distinct calendar periods aligned with the pandemic timeline in China. The distribution of samples and assays for each period is detailed in Supplementary Table 1. Overall, pooled data revealed significant temporal variations for the majority of primary semen and hormonal parameters (Table 1) across the study periods, laying the foundation for subsequent age-stratified analyses.

**Table 1:** Semen parameters values and reproductive hormone levels across the COVID-19 pandemic periods

Parameter	Pre-COVID-19 (2017–2019)	During COVID-19 (2020-2022.12.7)	Post-COVID-19 (Phase 1) (2022.12.8–2023)	Post-COVID-19 (Phase 2) (2024)	*p* value^a^
Semen Routine Parameters^b^		(*n* = 33,234)	(*n* = 12,869)	(*n* = 12,350)	
Semen Volume (mL)	N/A^c^	3.3(2.5–4.4)	3.2(2.3–4.2)	3.3(2.5–4.3)	< 0.001
Sperm Concentration (10^6^/mL)	N/A^c^	52.0 (28.0–86.0)	53.0 (26.0–93.0)	69.0 (35.0-116.0)	< 0.001
Total Sperm Count (10^6^)	N/A^c^	169.2 (85.5-284.8)	160.0 (76.0-288.0)	222.0 (108.0-382.6)	< 0.001
Progressive Motility (%)	N/A^c^	45.0(35.0–55.0)	49.0(36.0–61.0)	45.5(33.0-58.8)	< 0.001
Non-progressive Motility (%)	N/A^c^	9.0(6.0–12.0)	7.0(4.0–10.0)	7.4(5.1–10.1)	< 0.001
Immotility (%)	N/A^c^	46.0(34.0–57.0)	43.0(30.0–58.0)	46.4(32.8–58.7)	< 0.001
Sperm Morphology & Biochemical Parameters					
Normal Morphology (%)	4(4–6)	4(3–4)	4(3–4)	4(3–4)	< 0.001
AR (%)	15.83 (5.85–30.76)	11.50 (4.62–23.33)	11.43 (5.81–23.58)	12.21 (7.21–21.13)	< 0.001
Seminal Fructose (µmol)	40.64 (19.08–67.57)	41.93 (20.21–66.33)	37.96 (20.83–63.22)	50.05 (28.04–81.43)	< 0.001
Seminal Zinc (µmol)	7.85 (4.45–13.04)	6.88 (4.10-11.12)	6.71 (3.98–10.50)	7.34 (4.31–11.04)	< 0.001
Neutral α-glucosidase (mU)	47.36 (22.16–86.16)	34.61 (14.50-65.52)	34.26 (17.71–53.33)	45.16 (24.56–69.21)	< 0.001
Reproductive Hormones					
E2 (pg/mL)	23.80 (17.96-31.00)	23.00 (18.00–29.00)	21.33 (16.59-26.00)	23.08 (17.00-28.68)	< 0.001
FSH (mlU/mL)	5.46 (3.20-14.46)	5.40 (3.27–14.23)	5.04 (3.16–12.45)	5.28 (3.22–12.61)	0.470
LH (mlU/mL)	3.99 (2.6–6.77)	3.73 (2.5–6.01)	3.25 (2.19–5.46)	3.29 (2.28–5.43)	< 0.001
T (ng/mL)	4.46 (3.25–6.12)	4.36 (3.13–5.92)	4.31 (2.93–5.61)	4.19 (3.02–5.56)	< 0.001

Data are presented as median (interquartile range, IQR)

Identical medians do not preclude significant differences in distribution, and that statistical letters are based on pairwise comparisons of the full rank distributions

Abbreviations: IQR, interquartile range; CASA, Computer-Assisted Semen Analysis; AR, Acrosome Reaction; E2, estradiol; FSH, follicle-stimulating hormone; LH, luteinizing hormone; T, testosterone

^a^ p values are derived from Kruskal–Wallis test across the four time periods; pairwise comparisons were adjusted using Bonferroni correction (detailed in Supplementary Table 2)

^b^ Pre-COVID-19 data for routine semen parameters (volume, concentration, total count, motility) were obtained by manual counting and are not statistically comparable with later periods measured by Computer-Assisted Semen Analysis (CASA). These values are marked as N/A and are presented for descriptive context only

^c^ N/A: not applicable for statistical comparison due to methodological discontinuity (manual counting vs. CASA)

### Age-dependent divergence in semen parameter trends across the CASA-consistent period

**Fig. 2:**
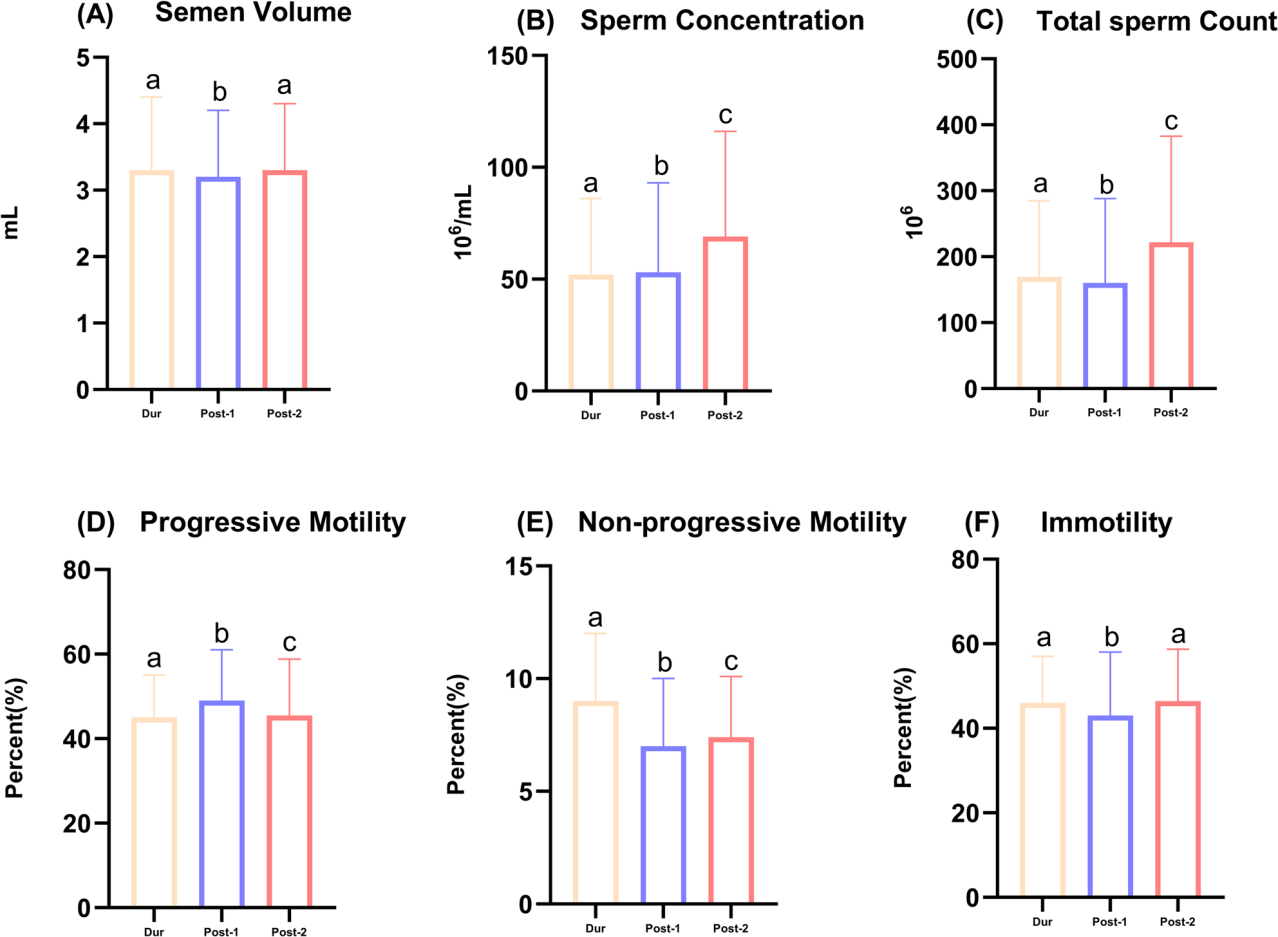
Temporal trends of conventional semen parameters across the COVID-19 pandemic periods. Data are presented as median with interquartile range (IQR). Panels show: **A** semen volume, **B** sperm concentration, **C** total sperm count, **D** progressive motility, **E** non-progressive motility, and **F** immotility. Different lowercase letters (a, b, c) indicate statistically significant differences between periods (*p* < 0.05, Kruskal–Wallis test with Bonferroni correction). Groups sharing the same letter are not significantly different. Note: Pre-COVID-19 data for these parameters were obtained by manual counting and are not statistically comparable with later CASA periods; therefore, only CASA-consistent periods (During, Post-1, Post-2) are shown in the statistical annotation and were included in the analysis. Abbreviations: COVID-19, coronavirus disease 2019; IQR, interquartile range; Dur, During COVID-19;Post-1, Post-COVID-19 Phase 1; Post-2, Post-COVID-19 Phase 2

Analysis of conventional semen parameters across the three CASA-consistent periods (During COVID-19, Post-Phase 1, and Post-Phase 2) revealed significant temporal trends for all parameters (all *p* < 0.001, Table 1). Specifically:

1. Semen volume transiently declined in Post-Phase 1 but recovered to During levels by Post-Phase 2.
2. Sperm concentration remained stable in Post-Phase 1, then increased significantly in Post-Phase 2.
3. Total sperm count dipped slightly in Post-Phase 1, followed by a substantial increase in Post-Phase 2.
4. Progressive motility improved in Post-Phase 1 but declined partially in Post-Phase 2, remaining above During levels. Conversely, non-progressive motility decreased in both post-pandemic phases, while immotility decreased in Post-Phase 1 but returned to near-During levels by Post-Phase 2 (Supplementary Tables 2 and Fig. 2).

**Fig. 3:**
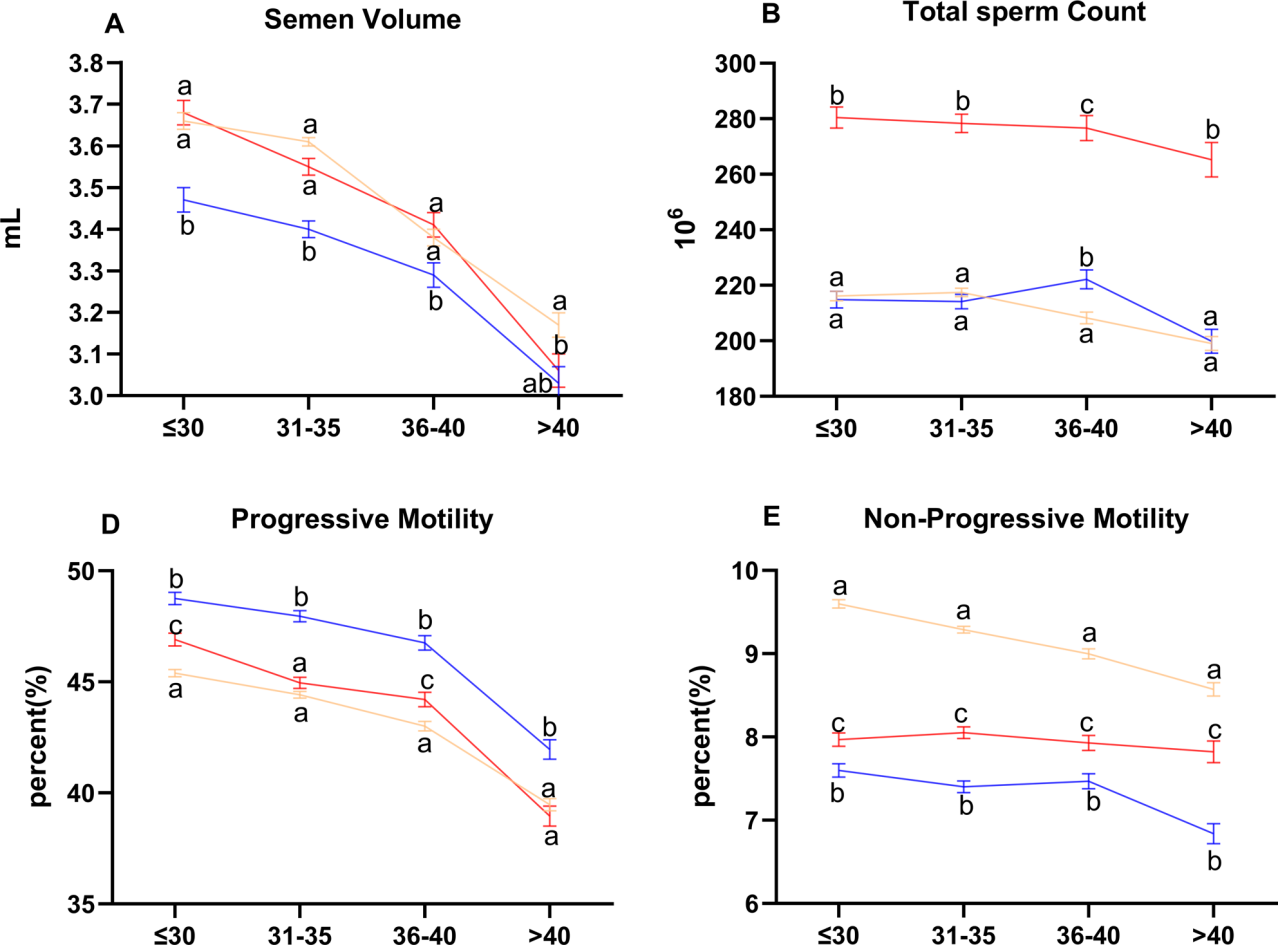
Age-stratified temporal trends of semen parameters with significant time × age interaction (generalized estimating equations, GEE). Data points represent estimated marginal means derived from generalized estimating equations (GEE) with time period, age group, and their interaction as fixed factors; error bars indicate standard error (SE). Panels display parameters with a significant time × age interaction (*p* < 0.05, Supplementary Table 3): **A** semen volume, **B** total sperm count, **D** progressive motility, and **E** non-progressive motility. Within each age group (≤ 30, 31–35, 36–40, > 40 years), different lowercase letters (a, b, c) denote significant differences between time periods (post-hoc pairwise comparisons with Bonferroni adjustment, *p* < 0.05). Groups sharing the same letter are not significantly different. Note that Pre-COVID-19 data for routine semen parameters (**A**–**E**) were measured by manual counting and were excluded from the GEE; therefore, only CASA-consistent periods (During, Post-1, Post-2) are shown for these parameters.Line colors: yellow = During COVID-19, blue = Post-COVID-19 Phase 1, red = Post-COVID-19 Phase 2

To investigate these dynamics further, we performed an age-stratified analysis. Generalized Estimating Equations (GEE) analysis revealed:

- Significant main effects of both time period and age group on all parameters (*p* < 0.001).
- Significant time-by-age interaction effects for semen volume, total sperm count, progressive motility, and non-progressive motility (all *p* < 0.05, Supplementary Table 3).
- Two distinct age-stratified patterns upon simple effects analysis (Supplementary Tables 4–5, Fig. 3).

In men aged ≤ 40 years old:

- Semen volume dipped transiently in Post-Phase 1 (*p* < 0.001) then recovered.
- Total sperm count rose steadily across phases (*p* < 0.001).
- Progressive motility peaked in Post-Phase 1 (*p* < 0.001) and remained above During levels in Post-Phase 2 (*p* < 0.001).

In men aged > 40 years old:

- Semen volume remained stable.
- Total sperm count was consistently lower than in younger men, though it increased in Post-Phase 2 (*p* < 0.001).
- Progressive motility increased in Post-Phase 1 (*p* < 0.001) but returned to During levels by Post-Phase 2.
- Trends for concentration and immotility mirrored younger groups (interaction *p* > 0.05), but absolute values were poorer.

### Sustained shifts in sperm morphology and age-modulated acrosomal function

**Fig. 4:**
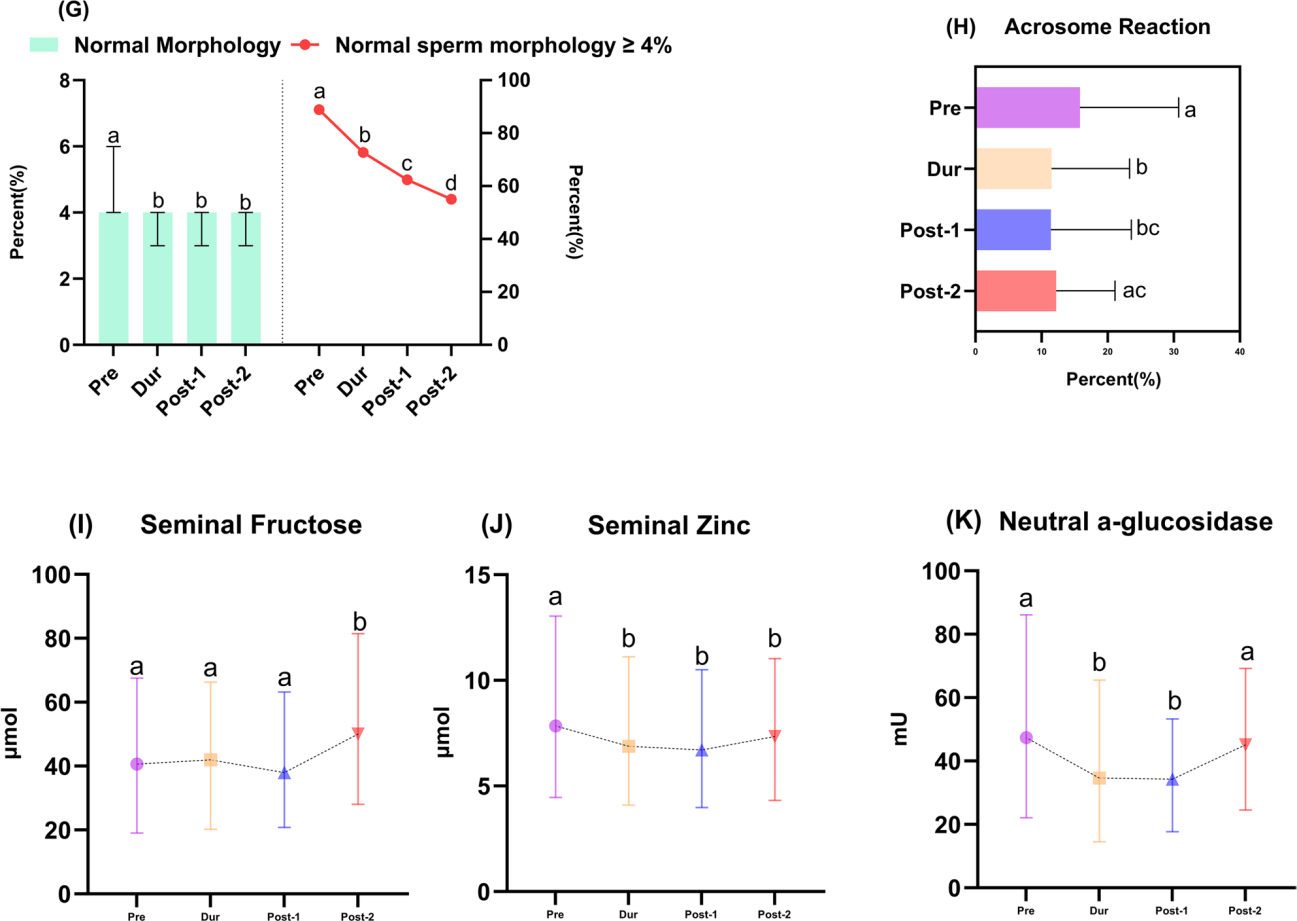
Temporal trends of sperm morphology, acrosomal function, and seminal plasma biochemistry across the COVID-19 pandemic periods. Data are presented as median with interquartile range (IQR). Panels show: **G** normal morphology and normal sperm morphology ≧ 4%, **H** acrosome reaction, **I** seminal fructose, **J** seminal zinc, and **K** neutral α-glucosidase. Different lowercase letters (a, b, c) indicate statistically significant differences between periods (*p* < 0.05, Kruskal–Wallis test with Bonferroni correction). Groups sharing the same letter are not significantly different. All parameters in this figure were measured using consistent laboratory methods throughout the entire study period (2017–2024). Abbreviations: COVID-19, coronavirus disease 2019; IQR, interquartile range; Pre, Pre-COVID-19;Dur, During COVID-19;Post-1, Post-COVID-19 Phase 1; Post-2, Post-COVID-19 Phase 2

The median normal sperm morphology rate remained at 4% across all periods, but the interquartile range (IQR) narrowed from 4 to 6% in the Pre-COVID-19 period to 3–4% in the During and both Post-COVID-19 phases, indicating a downward shift in distribution (Table 1). Accordingly, the proportion of men with normal morphology (≥ 4%) declined significantly from 88.9% pre-pandemic to 72.7% during the pandemic, 62.4% in Post-Phase 1, and 55.1% in Post-Phase 2 (all pairwise comparisons *p* < 0.001; Supplementary Tables 2 and 6). By Post-Phase 2, nearly half of the men exhibited normal morphology below the WHO diagnostic threshold (4%). Seminal plasma biochemistry exhibited parallel alterations: zinc and neutral α-glucosidase levels decreased during the pandemic (all *p* < 0.001). While α-glucosidase activity returned to pre-pandemic levels by Post-Phase 2, zinc levels remained depressed (Fig. 4J, K). Fructose levels increased in the late post-pandemic phase (*p* < 0.001; Fig. 4I).

### Temporal changes in reproductive hormone profiles

**Fig. 5:**
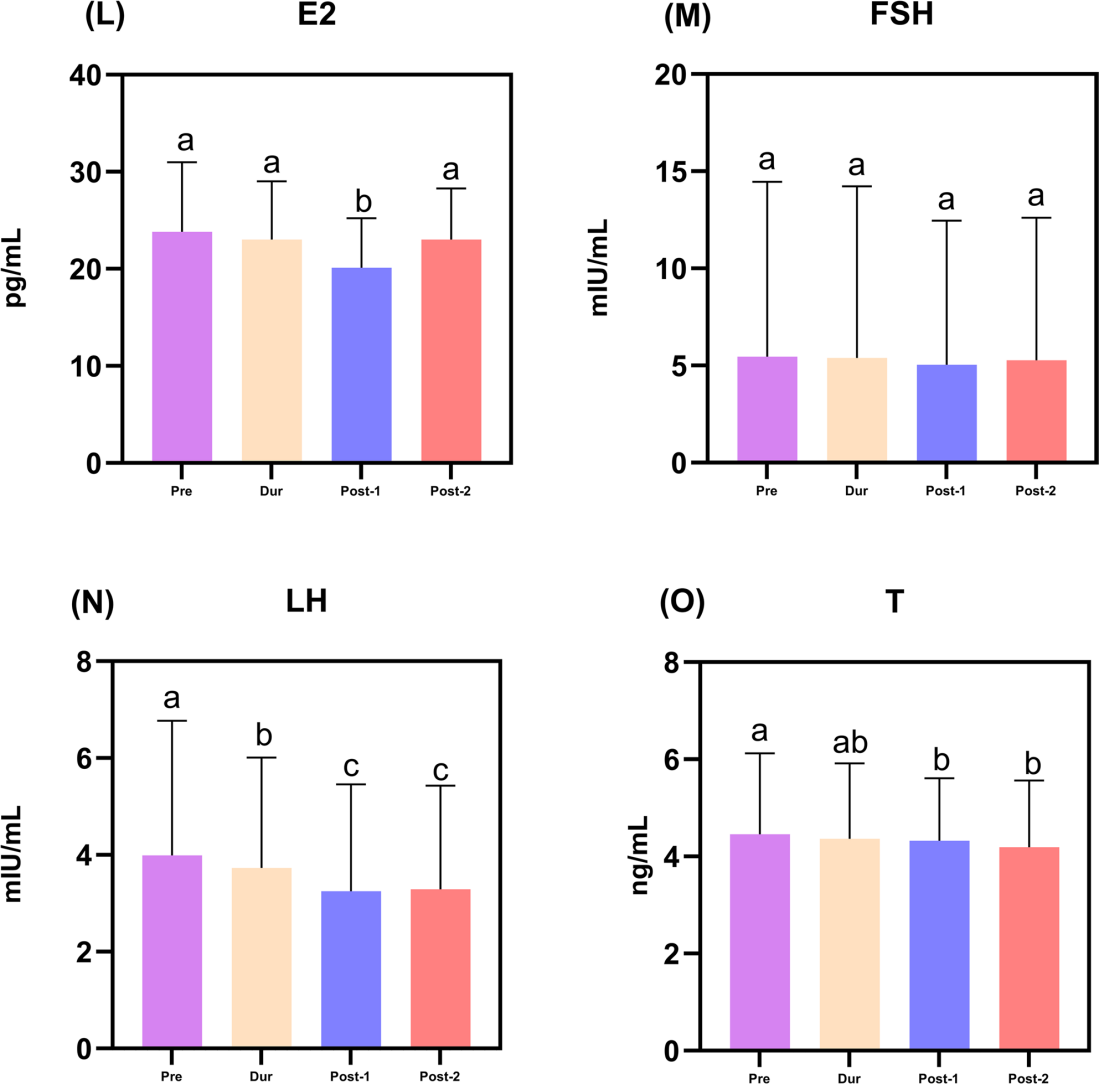
Temporal trends of reproductive hormone levels across the COVID-19 pandemic periods. Data are presented as median with interquartile range (IQR). Panels show: **L** estradiol (E2), **M** follicle-stimulating hormone (FSH), **N** luteinizing hormone (LH), and **O** testosterone (T). Different lowercase letters (a, b, c) indicate statistically significant differences between periods (*p* < 0.05, Kruskal–Wallis test with Bonferroni correction). Groups sharing the same letter are not significantly different. All parameters in this figure were measured using consistent laboratory methods throughout the entire study period (2017–2024). Abbreviations: COVID-19, coronavirus disease 2019;IQR, interquartile range; E2, estradiol; FSH, follicle-stimulating hormone; LH, luteinizing hormone; T, testosterone; Pre, Pre-COVID-19; Dur, During COVID-19;Post-1, Post-COVID-19 Phase 1; Post-2, Post-COVID-19 Phase 2

Reproductive hormone profiles showed distinct temporal changes (Fig. 5L-O, Supplementary Table 2). Estradiol (E2) levels showed only minor fluctuations. Luteinizing hormone (LH) exhibited a progressive decline from the pre-pandemic period through Post-Phase 1 (*p* < 0.001). Testosterone (T) levels showed a modest but statistically significant decline across the study periods. The median T concentration decreased from 4.46 ng/mL (IQR: 3.25–6.12) in the Pre-COVID-19 period to 4.19 ng/mL (IQR: 3.02–5.56) in Post-Phase 2 (absolute reduction: 0.27 ng/mL; relative reduction: 6.1%; *p* = 0.006 for Pre vs. Post-Phase 2; Supplementary Table 2). Pairwise comparisons revealed that T levels in Post-Phase 1 (median: 4.31 ng/mL, IQR: 2.93–5.61) were also significantly lower than pre-pandemic levels *p* = 0.011, whereas no significant difference was observed between the Pre-COVID-19 and During COVID-19 periods *p* = 0.591 (Table 1). Despite this gradual decline, the median T concentration in Post-Phase 2 remained well above the conventional clinical threshold for biochemical hypogonadism (typically < 3.0 ng/mL), suggesting that the observed reduction, while consistent, may not represent a widespread endocrine disorder at the population level, while follicle-stimulating hormone (FSH) remained stable. This pattern, declining T and LH with preserved FSH, is consistent with a profile often associated with secondary hypogonadism.

### Transient changes in anti-sperm antibody positivity

In contrast to the more sustained alterations in semen parameter values and hormone profiles, anti-sperm antibody positivity showed a transient temporal pattern. Positivity rates surged during the pandemic (*p* < 0.001 vs. Pre-COVID-19), remained elevated in Post-Phase 1, but significantly decreased by Post-Phase 2 (*p* < 0.001 vs. During COVID-19), approaching pre-pandemic levels (Supplementary Table 7).

## Discussion

From this large-scale retrospective analysis spanning eight years, we present unique longitudinal evidence on the temporal trends of male reproductive parameters across distinct phases of the COVID-19 pandemic. Our findings reveal divergent temporal trajectories across seminal and hormonal biomarkers within a clinically infertile population. While certain indicators (e.g., semen volume and progressive sperm motility) showed transient fluctuations, other key parameters, most notably normal sperm morphology and testosterone levels, demonstrated sustained downward shifts that failed to return to pre-pandemic baselines. This bifurcation pattern suggests that the constellation of stressors associated with the pandemic period may exert complex and lasting effects on the male reproductive system. However, whether these effects stem from direct viral infection, indirect psychosocial or lifestyle changes, or a combination thereof, cannot be determined from our period-based design. The persistent deficit in normal sperm morphology through the second post-pandemic year is a particularly notable finding that warrants further investigation.

Transient fluctuations in semen volume and anti-sperm antibody positivity are consistent with a temporary stress response. We interpret these observations through a multi-pathway framework, acknowledging that our period-comparison design precludes definitive disentanglement of causal factors. Specifically, the transient decrease in semen volume in Post-Phase 1 and the initial surge in anti-sperm antibody positivity are consistent with a temporary stress response. We hypothesize that this may be mediated by systemic inflammation, fever episodes, or psychological distress [7] associated with the pandemic environment, although direct evidence is lacking in this study.

Persistent impairments in sperm morphology and seminal zinc may reflect more enduring alterations in spermatogenic epithelium and prostate gland function [11–14], though this remains a hypothesis requiring histological or cellular validation. The partial recovery of neutral α-glucosidase, an epididymal marker, alongside a persistent morphology deficit [11], may implicate post-testicular dysfunction in the long-term alterations.

The observed endocrine pattern, declining LH and testosterone with preserved FSH, is consistent with H-P-G axis dysregulation, but its clinical interpretation remains uncertain. Furthermore, the observed hormonal pattern, a synchronized decline in LH and testosterone with preserved FSH [9, 15–18], is a functional profile resembling secondary hypogonadism. Whether H-P-G axis dysregulation represents a true hypogonadal state or an adaptive physiological response to systemic stress cannot be determined from our data. Such a pattern could also arise from chronic illness, sustained psychosocial stress, or other pandemic-related systemic disturbances, not solely from direct testicular damage.

These prolonged endocrine shifts share similarities with post-acute sequelae reported in other organ systems. It is plausible that reproductive endocrine dysfunction could represent a component of the broader post-pandemic health sequelae, though whether this is specifically attributable to viral infection or other pandemic-associated factors remains an open question.

Age is a critical modifier of reproductive parameter trajectories, with distinct temporal patterns observed between younger and older men, elucidated in our study by age-stratified trends. The significant time-by-age interactions indicate that reproductive parameters evolved differently across age groups throughout the pandemic phases; younger men exhibited more pronounced fluctuations in conventional semen quality. While the underlying mechanisms cannot be determined from this observational study, potential explanations for the more pronounced fluctuations in younger men include higher baseline physiological reactivity or differential exposure to stressors [19–21]. Conversely, older men’s delayed or attenuated recovery in semen volume, total sperm count and motility may involve age-related differences in HPG axis resilience [22], spermatogenic temporal patterns [23], or cohort-specific vulnerabilities [10, 13, 17, 24]. However, these remain speculations that cannot be directly tested with our observational data and require validation in mechanistic studies.

Beyond age, other modulating factors such as genetic variability may contribute to individual susceptibility. The heterogeneity in recovery trajectories also invites consideration of other modulating factors beyond age. Host genetic variability [13, 21, 25–30], particularly in genes related to viral entry (e.g., ACE2, TMPRSS2) or immune regulation, may also play a role. Although our study did not assess genetic factors, these should be included in future investigations into individual susceptibility to sustained reproductive alterations following environmental stressors. The patterns observed in our regional cohort highlight the need for future research integrating genetic and immunological data to move towards personalized risk assessment.

### Clinical significance of the observed shifts

Beyond statistical significance, the clinical relevance of our findings merits consideration. The sustained decline in normal sperm morphology is particularly noteworthy because the proportion of men with normal morphology below the WHO diagnostic threshold (4%) nearly quadrupled from 11.1% pre-pandemic to 44.9% in Post-Phase 2 (Supplementary Table 6). Previous longitudinal studies have demonstrated that each 1-percentage-point decrease in normal morphology is associated with a 5–10% reduction in natural conception rates [31]. Thus, the population-level shift toward poorer morphology—reflected in the narrowing interquartile range and the increased proportion below the threshold—may translate into non-negligible fertility implications, particularly for couples already at the margin of subfertility.

In contrast, the observed decline in serum testosterone, while statistically significant, remained above the conventional threshold for biochemical hypogonadism (3.0 ng/mL) in most individuals. This suggests that the observed endocrine shift is unlikely to represent a clinically overt hypogonadal state. However, given the continuous nature of testosterone’s physiological effects, even moderate reductions may have subtle impacts on libido, energy, or body composition of an individual [32], warranting attention in future prospective studies.

For seminal plasma biomarkers, the persistent depletion of zinc—a critical cofactor for sperm chromatin stability and antioxidant defense [33]—remains of uncertain clinical significance, as well-defined fertility thresholds for seminal zinc are lacking. This highlights the need for functional fertility outcome data in future research.

From a public health perspective, our findings underscore the critical importance of incorporating male reproductive health into long-term post-pandemic surveillance frameworks. Our data provide empirical evidence that major global health crises can be associated with prolonged and heterogeneous shifts in population-level male reproductive health markers at the population level. This reinforces the need to prioritise reproductive health within sustained post-pandemic health surveillance frameworks.

### Limitations of the study

Our findings should be interpreted in the context of several key limitations. First the retrospective, period-based study design cannot distinguish between the direct effects of SARS-CoV-2 infection and the indirect effects of pandemic-associated stressors (e.g., psychological distress, lifestyle changes, healthcare disruptions). The lack of individual-level data on infection status, vaccination history, and detailed clinical data (e.g., fever severity) represents a significant source of unmeasured confounding [34, 35]. Second, while laboratory protocols were standardized, the assessment of sperm morphology, though performed by trained personnel, is subject to inherent inter-observer variability. Although we cannot fully exclude the possibility of subtle procedural deviations, several measures were taken to ensure consistency throughout the study. Sperm morphology assessments were conducted by the same two experienced technicians throughout the study period, and regular intra-laboratory comparisons were performed. Additionally, the laboratory participated in a national external quality assessment (EQA) program from 2021 onwards. However, the absence of EQA data for the initial study period (2017–2020) represents a limitation. Future longitudinal studies should incorporate both internal and external quality controls from the outset to further strengthen data robustness.Third, the transition from manual counting to Computer-Assisted Semen Analysis (CASA) for routine semen parameters (concentration, motility, total count) introduced a methodological discontinuity between the Pre-COVID period and all subsequent periods. To eliminate this confounding factor, all statistical comparisons and temporal trend analyses for these parameters were strictly confined to the three periods employing consistent CASA methodology (During, Post-Phase 1, Post-Phase 2). Data from the Pre-COVID period (manual counting) are presented in tables for descriptive purposes only and were not included in any formal statistical testing or trend modeling. This methodological constraint does not undermine our core conclusions regarding: (i) age-stratified differential patterns within the CASA period, (ii) sustained shifts in sperm morphology and hormone profiles (which were measured by consistent methods throughout), or (iii) the transient surge in anti-sperm antibodies. While we cannot quantify the exact bias introduced by the method change, existing literature suggests that CASA tends to yield slightly higher estimates for motile sperm concentration compared to manual counting [36]. If uncorrected, such a bias would, if anything, lead to underestimation of any true decline from pre-pandemic levels; therefore, our restriction of all formal analyses to the CASA-consistent period represents a conservative approach. Future studies incorporating cross-calibrated methods are needed to directly assess long-term recovery relative to absolute pre-pandemic baselines. Fourth, our cohort consists of men presenting for infertility evaluation at a single regional center in China, which limits the generalizability of our findings to fertile populations or other geographical and ethnic groups. Fifth, the absence of data on sperm DNA fragmentation and other functional assays limits a more comprehensive assessment of sperm quality. Finally, while GEE identified significant time-by-age interactions, these observational associations cannot establish causality. Future prospective studies with pre-infection baselines, confirmed virological status, and detailed covariate collection are needed to validate these trajectories and elucidate underlying mechanisms.

## Conclusion

Our study identifies divergent long-term trajectories in male reproductive parameter values following the pandemic period, with persistent alterations in sperm morphology and testosterone levels being particularly notable. These trajectories were significantly modulated by age, underscoring the importance of age-stratified clinical monitoring. The findings highlight that the male reproductive system may exhibit domain-specific and heterogeneous responses to major population-level stressors. This warrants a clinical and public health framework that considers long-term reproductive health surveillance in the aftermath of global health crises. Future research should aim to integrate multi-omics approaches with clinical phenotyping to clarify the specific roles of viral infection, systemic stress, and host factors in shaping post-stress reproductive trajectories, ultimately informing personalized fertility counseling and preservation strategies. Importantly, as our study compares population-level temporal trends rather than individual infection status, the relative contributions of direct viral effects and indirect pandemic-associated stressors remain to be disentangled in future research. 

## Supplementary Information

Supplementary Material 1.

## Data Availability

The datasets generated and/or analysed during the current study are not publicly available due to privacy and ethical restrictions related to human subject research but are available from the corresponding author on reasonable request.

## Abbreviation

GEE: Generalized estimating equations
ACE2: angiotensin-converting enzyme 2
TMPRSS2: transmembrane serine protease 2
AR: Acrosome reaction rate
E2: Serum estradiol
FSH: follicle-stimulating hormone
LH: luteinizing hormone
T: testosterone
CASA: Computer-Assisted Semen Analysis
PSA-FITC: fluorescein isothiocyanate-labeled Pisum sativum agglutinin
IQR: interquartile range
HPG: hypothalamic-pituitary-gonadal
