# Supplementary material for: Divergent long-term trends in semen quality and reproductive hormones across the COVID-19 pandemic era in infertile men: an age-stratified retrospective study
Source: Basic Clin Androl. 2026 Mar 26;36:10. doi: 10.1186/s12610-026-00309-1 (PMC13020235; doi:10.1186/s12610-026-00309-1)
Supplement: Supplementary file 1 — Supplementary Material 1. [file 12610_2026_309_MOESM1_ESM.docx]

Supplementary Table 1. Sample sizes for each assay across the study periods

| **Assay** | **Pre-COVID-19** | **During COVID-19** | **Post-COVID-19**  **(Phase 1)** | **Post-COVID-19**  **(Phase 2)** |
| --- | --- | --- | --- | --- |
| Sperm Morphology | 32,211 | 31,848 | 13,132 | 12,934 |
| Acrosome Reaction | 424 | 1,110 | 613 | 1,492 |
| Seminal Fructose | 2,713 | 946 | 567 | 1,304 |
| Seminal Zinc | 2,483 | 937 | 549 | 1,304 |
| Neutral α-glucosidase | 2,724 | 947 | 568 | 1,309 |
| E2 | 1,298 | 1,388 | 478 | 453 |
| FSH | 1,589 | 1,442 | 512 | 482 |
| LH | 1,479 | 1,423 | 483 | 821 |
| T | 1,483 | 1,436 | 507 | 470 |

Note: Sample sizes vary across assays due to the availability of residual samples and clinical indications at each visit. All assays were performed using consistent methodologies throughout the entire study period.

Abbreviations:E2,estradiol;FSH,folliclestimulating hormone;LH, luteinizing hormone; T, testosterone.

Supplementary Table 2. Pairwise comparisons of parameters values across pandemic periods (Bonferroni-adjusted p values)

| **Parameter** | **Pre vs. Dur** | **Pre vs. Post-1** | **Pre vs. Post-2** | **Dur vs. Post-1** | **Dur vs. Post-2** | **Post-1**  **vs.Post-2** |
| --- | --- | --- | --- | --- | --- | --- |
| Semen Routine (CASA) |  |  |  |  |  |  |
| Semen Volume (mL) | N/A^a^ | N/A^a^ | N/A^a^ | <0.001 | 0.467 | <0.001 |
| Sperm Concentration  (10^6^/mL) | N/A^a^ | N/A^a^ | N/A^a^ | 0.024 | <0.001 | <0.001 |
| Total sperm Count (10^6^) | N/A^a^ | N/A^a^ | N/A^a^ | 0.003 | <0.001 | <0.001 |
| Progressive Motility (%) | N/A^a^ | N/A^a^ | N/A^a^ | <0.001 | <0.001 | <0.001 |
| Non-prog.Motility (%) | N/A^a^ | N/A^a^ | N/A^a^ | <0.001 | <0.001 | <0.001 |
| Immotility (%) | N/A^a^ | N/A^a^ | N/A^a^ | <0.001 | 0.157 | <0.001 |
| Morphology&Biochemical |  |  |  |  |  |  |
| Normal Morphology (%) | <0.001 | <0.001 | <0.001 | <0.001 | <0.001 | <0.001 |
| Acrosome Reaction (%) | <0.001 | 0.048 | 0.089 | 0.718 | 0.029 | 1.000 |
| Seminal Fructose (μmol) | 1.000 | 1.000 | <0.001 | 1.000 | <0.001 | <0.001 |
| Seminal Zinc (μmol) | <0.001 | <0.001 | <0.001 | 1.000 | 1.000 | 0.602 |
| Neutral  α-glucosidase (mU) | <0.001 | <0.001 | 0.087 | 1.000 | <0.001 | <0.001 |
| Reproductive Hormones |  |  |  |  |  |  |
| E2 (pg/mL) | 0.243 | <0.001 | 0.582 | 0.001 | 1.000 | 0.019 |
| FSH (mlU/mL) | 1.000 | 1.000 | 1.000 | 0.880 | 1.000 | 1.000 |
| LH (mlU/mL) | 0.034 | <0.001 | <0.001 | 0.006 | 0.003 | 1.000 |
| T (ng/mL) | 0.591 | 0.011 | 0.006 | 0.329 | 0.204 | 1.000 |
| Other Outcomes |  |  |  |  |  |  |
| ASA Positive (%) | <0.001 | <0.001 | <0.001 | <0.001 | <0.001 | <0.001 |
| Normal Morph. ≥4% (%) | <0.001 | <0.001 | <0.001 | <0.001 | <0.001 | <0.001 |

Note: p values are derived from pairwise comparisons following Kruskal–Wallis test (for continuous variables) or Chisquared test (for categorical variables: ASA positivity, normal morphology ≥4%), adjusted using Bonferroni correction. ^a^N/A: not applicable due to methodological discontinuity (manual counting vs. CASA). Abbreviations: ASA, antisperm antibody; CASA, ComputerAssisted Semen Analysis; Dur, During COVID-19; Post1, Post-COVID-19 Phase 1; Post2, Post-COVID-19 Phase 2; Pre,Pre-COVID-19; E2,estradiol; FSH,folliclestimulating hormone; LH,luteinizing hormone; T, testosterone.

Supplementary Table 3. Generalized Estimating Equations (GEE) Assessing the Main Effects of Time and Age Group, and their Interaction on Semen and Hormone Parameters

| **Parameter** | **Time Effect** |  | **Age Effect** |  | **Time×Age**  **Group**  **interaction** |  |
| --- | --- | --- | --- | --- | --- | --- |
|  | Waldχ² | p | Waldχ² | p | Waldχ² | p |
| Semen Routine (CASA only) |  |  |  |  |  |  |
| Semen Volume (mL)^a^ | 79.16 | <0.001 | 456.28 | <0.001 | 16.05 | 0.013 |
| Sperm Concentration^a^  (10^6^/mL) | 906.33 | <0.001 | 119.02 | <0.001 | 11.19 | 0.083 |
| Total sperm Count (10^6^)^b^ | 785.30 | <0.001 | 30.49 | <0.001 | 15.11 | 0.019 |
| Progressive Motility (%)^c^ | 273.61 | <0.001 | 624.48 | <0.001 | 13.47 | 0.036 |
| Non-prog.Motility (%)^c^ | 1123.35 | <0.001 | 66.57 | <0.001 | 29.91 | <0.001 |
| Immotility (%)^c^ | 72.01 | <0.001 | 739.87 | <0.001 | 9.15 | 0.166 |
| Morphology&Biochemical |  |  |  |  |  |  |
| Normal Morphology (%)^c^ | 13516.05 | <0.001 | 4.78 | 0.189 | 12.80 | 0.172 |
| Acrosome Reaction (%)^c^ | 18.10 | <0.001 | 1.71 | 0.635 | 16.09 | 0.065 |
| Seminal Fructose (μmol)^b^ | 74.25 | <0.001 | 53.29 | <0.001 | 12.89 | 0.168 |
| Seminal Zinc (μmol)^b^ | 82.27 | <0.001 | 7.00 | 0.072 | 6.72 | 0.666 |
|  |  |  |  |  |  |  |
| Neutral  α-glucosidase (mU)^a^ | 120.40 | <0.001 | 18.41 | <0.001 | 9.96 | 0.354 |
| Reproductive Hormones |  |  |  |  |  |  |
| E2 (pg/mL)^a^ | 54.88 | <0.001 | 3.77 | 0.288 | 8.65 | 0.470 |
| FSH (mlU/mL)^b^ | 5.00 | 0.172 | 0.61 | 0.894 | 9.23 | 0.416 |
| LH (mlU/mL)^b^ | 27.30 | <0.001 | 4.08 | 0.253 | 7.77 | 0.558 |
| T (ng/mL)^a^ | 22.69 | <0.001 | 3.67 | 0.299 | 6.335 | 0.706 |

Note: Generalized estimating equations (GEE) with an exchangeable correlation structure were used to account for repeated measurements. Models included time period, age group (≤30, 31–35, 36–40, >40 years), and their interaction as fixed factors, with significance assessed by Wald χ² tests (p < 0.05). Based on outcome distributions, we applied:

^a^Gamma regression with log link for strictly positive skewed variables

^b^Tweedie regression with log link for outcomes with a mass at zero

^c^Fractional logit regression with logit link for percentage outcomes

All routine semen parameters (concentration, motility, total count) were analyzed exclusively across the three CASAconsistent periods (During, Post1, Post2) due to a methodological transition from manual counting.

Abbreviations:E2, estradiol; FSH, folliclestimulating hormone; LH, luteinizing hormone; T, testosterone.

Supplementary Table 4. Estimated marginal means of parameters with significant time × age interaction (GEE)

| **Time Group** | **Age Group** | **Semen**  **Volume (mL)** | **Total**  **Sperm**  **Count (10^6^)** | **Progressive**  **Motility (%)** | **Non-prog.**  **Motility (%)** |
| --- | --- | --- | --- | --- | --- |
| Pre | ≤30 | N/A^a^ | N/A^a^ | N/A^a^ | N/A^a^ |
|  | 31-35 | N/A^a^ | N/A^a^ | N/A^a^ | N/A^a^ |
|  | 36-40 | N/A^a^ | N/A^a^ | N/A^a^ | N/A^a^ |
|  | >40 | N/A^a^ | N/A^a^ | N/A^a^ | N/A^a^ |
| During | ≤30 | 3.66±0.02 | 216.18±1.78 | 45.4±0.2 | 9.6±0.1 |
|  | 31-35 | 3.61±0.01 | 217.43±1.54 | 44.4±0.2 | 9.3±0.1 |
|  | 36-40 | 3.38±0.02 | 208.23±2.12 | 43.0±0.2 | 9.0±0.1 |
|  | >40 | 3.17±0.03 | 199.01±2.57 | 39.5±0.3 | 8.6±0.1 |
| Post-1 | ≤30 | 3.47±0.03 | 214.80±3.09 | 48.8±03 | 7.6±0.1 |
|  | 31-35 | 3.40±0.02 | 214.16±2.61 | 48.0±0.3 | 7.4±0.1 |
|  | 36-40 | 3.29±0.03 | 222.19±3.41 | 46.8±0.3 | 7.5±0.1 |
|  | >40 | 3.03±0.04 | 199.82±4.33 | 42.0±0.4 | 6.8±0.1 |
| Post-2 | ≤30 | 3.68±0.03 | 280.47±3.87 | 46.9±0.3 | 8.0±0.1 |
|  | 31-35 | 3.55±0.02 | 278.32±3.33 | 45.0±0.3 | 8.1±0.1 |
|  | 36-40 | 3.41±0.03 | 276.64±4.47 | 44.2±0.3 | 7.9±0.1 |
|  | >40 | 3.06±0.04 | 265.24±6.23 | 39.0±0.5 | 7.8±0.1 |

Data are estimated marginal means ± standard error derived from generalized estimating equations (GEE) with time period, age group, and their interaction as fixed factors. Only parameters with a significant time × age interaction (p < 0.05, see Supplementary Table 3) are shown. ^a^N/A: Pre-COVID19 values for routine semen parameters are not available owing to a methodological transition from manual counting to CASA and were excluded from the CASAperiod GEE analysis.

Supplementary Table 5. Simple effects analysis: pairwise comparisons across time periods within each age group for parameters with significant time × age interaction (GEE)

| **Parameter** | **Age Group** | **Pre vs.**  **Dur** | **Pre vs. Post-1** | **Pre vs. Post-2** | **Dur vs. Post-1** | **Dur vs. Post-2** | **Post-1 vs. Post-2** |
| --- | --- | --- | --- | --- | --- | --- | --- |
| Semen  Volume (mL) | ≤30 | N/A^a^ | N/A^a^ | N/A^a^ | MD=0.19 p<0.001 | MD=-0.02  p=1.000 | MD=-0.22 p<0.001 |
|  | 31-35 | N/A^a^ | N/A^a^ | N/A^a^ | MD=0.21  p<0.001 | MD=0.06  P=0.052 | MD=-0.15  p<0.001 |
|  | 36-40 | N/A^a^ | N/A^a^ | N/A^a^ | MD=0.09 p=0.031 | MD=-0.03  p=1.000 | MD=-0.12  p=0.013 |
|  | >40 | N/A^a^ | N/A^a^ | N/A^a^ | MD=0.14  p=0.004 | MD=0.11 p=0.056 | MD=-0.03  p=1.000 |
| Total  Sperm  Count (10^6^) | ≤30 | N/A^a^ | N/A^a^ | N/A^a^ | MD=1.38 p=1.000 | MD=-64.30 p<0.001 | MD=-65.68  p<0.001 |
|  | 31-35 | N/A^a^ | N/A^a^ | N/A^a^ | MD=3.27 p=0.810 | MD=-60.90 p<0.001 | MD=-64.17  p<0.001 |
|  | 36-40 | N/A^a^ | N/A^a^ | N/A^a^ | MD=-13.96 p=0.001 | MD=-68.41 p<0.001 | MD=-54.45  p<0.001 |
|  | >40 | N/A^a^ | N/A^a^ | N/A^a^ | MD=-0.81 p=1.000 | MD=-66.23p<0.001 | MD=-65.42p<0.001 |
| Progressive Motility (%) | ≤30 | N/A^a^ | N/A^a^ | N/A^a^ | MD=-3.36 p<0.001 | MD=-1.51  p<0.001 | MD=1.85 p<0.001 |
|  | 31-35 | N/A^a^ | N/A^a^ | N/A^a^ | MD=-3.54 p<0.001 | MD=-0.53  p=0.196 | MD=3.01  p<0.001 |
|  | 36-40 | N/A^a^ | N/A^a^ | N/A^a^ | MD=-3.75 p<0.001 | MD=-1.19 p=0.009 | MD=2.56p<0.001 |
|  | >40 | N/A^a^ | N/A^a^ | N/A^a^ | MD=-2.50 p<0.001 | MD=051  p=1.000 | MD=3.00  p<0.001 |
| Non-prog.  Motility (%) | ≤30 | N/A^a^ | N/A^a^ | N/A^a^ | MD=2.00 p<0.001 | MD=1.63 p<0.001 | MD=-0.38 p<0.001 |
|  | 31-35 | N/A^a^ | N/A^a^ | N/A^a^ | MD=1.90 p<0.001 | MD=1.25  p<0.001 | MD=-0.65 p<0.001 |
|  | 36-40 | N/A^a^ | N/A^a^ | N/A^a^ | MD=1.53  p<0.001 | MD=1.07 p<0.001 | MD=-0.46  p=0.001 |
|  | >40 | N/A^a^ | N/A^a^ | N/A^a^ | MD=1.73 p<0.001 | MD=0.75 p<0.001 | MD=-0.98 p<0.001 |

Note: Mean differences (MD) and p values are derived from post-hoc pairwise comparisons of estimated marginal means within each age group, using generalized estimating equations (GEE) with Bonferroni adjustment for multiple comparisons. ^a^N/A: Pre-COVID19 data not included in GEE due to methodological discontinuity (manual counting vs. CASA). Abbreviations: Dur, During COVID-19; Post1, Post-COVID-19 Phase 1; Post2, Post-COVID-19 Phase 2; Pre, Pre-COVID-19.

Supplementary Table 6. Distribution of normal sperm morphology (<4% vs. ≥4%) and intergroup comparisons

| **Normal Sperm Morphology** | **Pre-COVID-19** | **During COVID-19** | **Post-COVID-19**  **(Phase 1)** | **Post-COVID-19**  **(Phase 2)** | **p value^a^** |
| --- | --- | --- | --- | --- | --- |
| <4% (n) | 3,576 | 8,680 | 4,950 | 5,813 |  |
| ≧4% (n) | 28,635 | 23,168 | 8,207 | 7,121 |  |
| Proportion ≧4% (%) | 88.90 | 72.74 | 62.38 | 55.06 | <0.001 |

^a^ Chisquare test across the four periods;Post hoc pairwise comparisons were corrected using the Bonferroni method (adjusted α=0.0083), and all paired comparisons yielded P<0.001. Detailed values are presented in Supplementary Table 2.

Supplementary Table 7. Antisperm antibody positivity and intergroup comparisons

| **Antisperm Antibody** | **Pre-COVID-19** | **During COVID-19** | **Post-COVID-19 (Phase 1)** | **Post-COVID-19 (Phase 2)** | **p value**^a^ |
| --- | --- | --- | --- | --- | --- |
| Positive (n) | 1,415 | 2,842 | 865 | 697 |  |
| Negative (n) | 25,078 | 24,003 | 9,606 | 9,560 |  |
| Positive (%) | 5.34 | 10.58 | 8.26 | 6.79 | <0.001 |

^a^ Chisquared test across the four periods; Post hoc pairwise comparisons were corrected using the Bonferroni method (adjusted α=0.0083), and all paired comparisons yielded P<0.001. Detailed values are presented in Supplementary Table 2.
